# Nonshared environment: Real but random

**DOI:** 10.1002/jcv2.12229

**Published:** 2024-02-22

**Authors:** Robert Plomin

**Affiliations:** ^1^ King's College London Institute of Psychiatry, Psychology and Neuroscience London UK

**Keywords:** clones, endogenous noise, genetics, nonshared environment, parenting, randomness

## Abstract

**Background:**

In the excitement about genomics, it is easy to lose sight of one of the most important findings from behavioural genetics: At least half of the variance of psychopathology is caused by environmental effects that are not shared by children growing up in the same family, which includes error of measurement. However, a 30‐year search for the systematic causes of nonshared environment in a line‐up of the usual suspects, especially parenting, has not identified the culprits.

**Method:**

I briefly review this research, but primarily consider the conceptual framework of the search for ‘missing’ nonshared environmental effects.

**Results:**

The search has focused on exogenous events like parenting, but nonshared environment might not be caused by anything we would call an event. Instead, it might reflect endogenous processes such as noisy biological systems (such as somatic mutations and epigenetics) or, at a psychological level, idiosyncratic subjective perceptions of past and present experiences, which could be called nonshared environmental *experience* to distinguish it from exogenous events. Although real, nonshared environment might be random in the philosophy of science sense of being unpredictable, even though it can have stable effects that predict subsequent behaviour.

**Conclusion:**

I wade into the weeds of randomness and suggest that this so‐called ‘gloomy prospect’ might not be so gloomy.

**Key Points**

- Nonshared environmental effects are real—this is how nongenetic factors account for half of the variance in psychopathology.
- After 3 decades of research, exogenous predictors of nonshared are only able to predict 1%–2% of the variance of psychopathology.
- Complex biological and psychological systems can create random (unpredictable) endogenous nonshared environmental effects independent of exogenous events.
- The conclusion that nonshared environment is real but random may not be a gloomy prospect: We can understand how nonshared environment works even if we cannot predict it.

## INTRODUCTION

Early behavioural genetic research was like a figure‐ground illusion in that researchers looked for genetic influence as the foreground; the environment was the background residual variance not explained by genetics (Plomin, 2023). The goal was to disentangle nature and nurture in familial resemblance. Two widely used designs are twin studies and adoption studies (Knopik et al., 2017). Twin studies compare the resemblance of identical and fraternal twins. To the extent that genetics accounts for family resemblance for a trait, identical twins will be more similar than fraternal twins because identical twins are at least twice as similar genetically as fraternal twins. Adoption designs assess genetic influence by studying the resemblance of family members who share nature but not nurture, such as birth parents and their adopted‐away offspring. In both designs, to the extent that familial resemblance cannot be explained by nature, it is attributed to nurture or residual environmental factors. In modern model‐fitting analyses, generally referred to as ACE models, these components of variance are denoted A (additive genetic influence) and C (common environmental influence shared by family members). Residual variance not explained by A or C is labelled as E.

This figure‐ground illusion flipped for me in the summer of 1976 when I read a newly published book by John Loehlin and Robert Nichols, *Heredity, Environment, and Personality: A Study of 850 Sets of Twins* (Loehlin & Nichols, 1976). They confirmed the importance of genetics, which accounted for about 40% of the variance of personality and about 50% for general cognitive ability, and they added many novel analyses such as analyses of early environment, comparisons between twins and nontwins, and multivariate genetic analyses. What struck me most was their conclusion about the environment:

Environment carries substantial weight in determining personality— it appears to account for at least half the variance— but that environment is one for which twin pairs are correlated close to zero… We seem to see environmental effects that operate almost randomly with respect to the sorts of variables that psychologists (and other people) have traditionally deemed important.

(Loehlin & Nichols, 1976, p. 92)

In other words, growing up in the same family does not make children similar environmentally. As always, one can find earlier passing references to this phenomenon (e.g., Woodworth, 1941; Wright, 1920), but Loehlin and Nichols highlighted the issue.

Perhaps because Loehlin and Nichol's conclusion about environmental influence was somewhat buried in a book with many novel findings, it had little impact on the field. In 1981, David Rowe and I highlighted the importance of this finding in an article that gave the phenomenon the label of nonshared environment (Rowe & Plomin, 1981). The environment is important, but it is not the environment shared by family members that makes them similar, as connoted by the word ‘nurture’. We called it ‘nonshared’ environment (NSE) because it does not make children growing up in the same family similar.

In 1987, Denise Daniels and I summarised the evidence for the importance of nonshared environment for psychopathology, personality and cognitive ability (Plomin & Daniels, 1987a). The article, *‘Why are children in the same family so different from one another?’,* was published with 32 commentaries in *Behavioural and Brain Sciences*, none of which disagreed with the conclusion that children growing up in the same family are very different, especially for psychopathology and personality, although disagreement abounded about the causes of these differences. The finding that most environmental effects on psychopathology and personality are not shared by children growing up in the same family has become one of the top replicated findings in behavioural genetics (Plomin et al., 2016; Turkheimer, 2000). The focus of the present article is on psychopathology; issues of shared and nonshared environment are more complex for cognitive abilities (Plomin & Kawakami, 2023). By ‘psychopathology,’ I refer to both dimensional and dichotomous (diagnostic) approaches, although dimensional approaches are more amenable to assessing variance explained in a population; dichotomous approaches need to resort to liability models that assume a continuum underlying the dichotomy.

At this point, the standard caveat should be mentioned that behavioural genetic research describes ‘what is’; it does not predict ‘what could be’. That is, research showing that nearly all environmental influence on psychopathology is nonshared describes the origins of individual differences in a particular population at a particular time; it does not predict what could be, including the effects of interventions or treatments. Because causes and cures are not necessarily related, a novel intervention or treatment for psychopathology, whether shared or nonshared, could have a major impact.

Our response to the 32 commentaries was called ‘Children in the same family are very different, but why?’ (Plomin & Daniels, 1987b). We listed categories of environmental influences that can cause children in the same family to differ (Table 1). Note that these are categories of environmental events that could have nonshared environmental effects, which is a separate issue from whether the event itself is shared or not shared by siblings. For example, parental divorce happens to both siblings, but its effects might be different for each sibling. It should also be noted that genetics predicts that siblings other than identical twins differ for heritable traits because their 0.50 coefficient of genetic relatedness means that they are 50% different genetically for segregating alleles.

**TABLE 1:** Categories of nongenetic factors that could cause children in the same family to differ (adapted from Plomin & Daniels, 1987a).

Category	Example
Error of measurement	Test‐retest unreliability
Nonshared environment	
Nonsystematic	Accidents, illnesses, trauma
Systematic	
Family composition	Birth order, gender
Sibling interaction	Differential sibling relationships
Parental treatment	Differential parenting
Extrafamilial networks	Peer groups, teachers, social media

We outlined three steps necessary to establish whether a putative measure of the environment has nonshared environmental effects. Using parenting as the candidate targeted most by researchers, the first step is to ask whether parents treat siblings differently. This requires studying more than one child per family. Although twins were central to behavioural genetic designs that seek to separate nature from nurture, research in developmental psychology rarely included more than one child per family. The second step is to demonstrate that differences in parenting are associated with differences in siblings' outcomes. The third step follows from recognising that genetics can contribute to these associations because siblings are 50 percent different genetically and parents might be responding to these differences: show that differences in parenting predict differences in siblings' outcomes when genetics is controlled. That is, calling a measure like parenting an environmental measure does not make it so (Kendler & Baker, 2007).

The simplest design is to ask what makes identical twins different (Asbury et al., 2006; Pike et al., 1996). This design jumps directly to the third step because associations between differences in parenting towards identical twins and the twins' behavioural differences can only be due to nonshared environmental influences. Error of measurement also makes identical twins differ, but this source of variance does not covary between parenting and twins' behaviour. Although asking what makes identical twins different is a sharp scalpel for dissecting nonshared environment, the design is not tethered to total variance. It only examines within‐family variance and does not address the contribution of nonshared environment to total variance. However, it is an ideal design for idiographic investigation, which, as discussed later, may be necessary to understand random nonshared environmental processes. Other genetic designs, such as the full twin method that compares identical and nonidentical twins, use multivariate model‐fitting approaches to allocate total variance to these components of variance and their covariance.

In 1987, few studies had been reported that made it to the third step of asking if differences in parenting relate to differences in siblings' outcomes when genetics is controlled (Plomin & Daniels, 1987a). Reviews of early studies found that candidates for nonshared environment often falter at the second step and stumble at the third step (Turkheimer & Waldron, 2000), accounting for about 1%–2% of the variance. This was also the conclusion of a 10‐year longitudinal study of 700 families that included twins, full siblings, half siblings and adoptive siblings, called Nonshared Environment in Adolescent Development, which focused on the search for nonshared environmental influences (Reiss et al., 2000). Studies that pass the third step usually involve the same rater; typically the parent rates both their parenting and their children's behaviour contemporaneously (e.g., Nikstat & Riemann, 2023). However, effect sizes are modest and disappear altogether across raters (Nikstat & Riemann, 2023) and across time (Pike et al., 1996).

This was the result of a recent ACE model‐fitting analysis of longitudinal data from preschool to adulthood for 4000 pairs of twins using multiple environmental measures and multiple behaviour problems as rated by parents, teachers and the twins themselves (Gidziela, Malanchini, et al., 2022). For example, the cumulative nonshared environmental effect of parent ratings of multiple environmental measures in preschool, childhood and adolescence predicted only 0.2% of the nonshared environmental variance of twins' self‐reported behaviour problems at age 21. The authors concluded: ‘The missing NSE gap between variance explained by measured environments and total NSE variance is large’ (Gidziela, Malanchini, et al., 2022). A recent meta‐analysis of studies using ‘quasi‐experimental’ designs also showed that the association between childhood maltreatment and mental health problems is small, accounting for about 2% of the variance (Baldwin et al., 2023).

Absence of evidence is not evidence of absence and researchers will continue to look for systematic sources of nonshared environment (e.g., Gjerde et al., 2021). My goal is not to discourage or disparage these studies, nor to say that such studies have explained none of the nonshared environmental variance, but rather to point out that, after 30 years of research, the missing nonshared environment gap is large (Plomin, 2011).

In our 1987 article, we raised what we called ‘the gloomy prospect … that the salient environment might be unsystematic, idiosyncratic, or serendipitous events’—in a word, random (Plomin & Daniels, 1987b, p. 8). Although we suggested that it ‘makes sense to start the search by looking for systematic sources of variance’, the subsequent failure to find measured environmental effects responsible for nonshared environmental influence made the gloomy prospect look more likely. For example, a decade later, after a meta‐analytic review of early research on this topic, Eric Turkheimer concluded that ‘The gloomy prospect is true’ (Turkheimer, 2000, p. 163). Now, 2 decades later, it is time to take the gloomy prospect seriously. The purpose of the present article is not to provide a systematic review of the evidence for nonshared environmental influence or of attempts to identify systematic sources of nonshared environment, but rather to consider randomness in the context of the conceptual framework of the search for missing NSE. I suggest that the gloomy prospect might not be so gloomy.

## RANDOMNESS

Randomness is a complex topic in the philosophy of science, but a useful definition is that randomness is unpredictability (Eagle, 2005). The words *randomness* and *chance* differ in that randomness refers to an event's unpredictability whereas chance refers to the probability of the event happening. For example, the outcome of a spinning roulette wheel is unpredictable (random) although the probability of a particular outcome such as a bet on a single number can be predicted (chance). Luck is quite different; it implies an illusion of control over random events (Krantz, 1998; Wgenaar & Kerenm, 1988).

As indicated in Table 1, an obvious source of randomness is major events such as accidents and illness. Although biographies and autobiographies often point to such chance events as tipping points to explain why siblings are so different (Dunn & Plomin, 1990), research suggests that they are not likely to be a major cause of nonshared environmental effects in the population. For example, an idiographic study of adult identical twin pairs discordant for lifetime major depressive disorder interviewed the twins about their life experiences (Kendler & Halberstadt, 2013). Only one pair pinned their depression discordance on a single event, the depressed twin's motorcycle accident in adolescence. The authors concluded that, contrary to their expectation, ‘It was rare for one major life event to cause the life course of the twins to diverge’ (p. 980).

Such examples are analogous to major gene effects on disorders. Thousands of single‐gene disorders are known that have dramatic effects on affected individuals, but single‐gene disorders are rare and account for negligible variance in the population (Knopik et al., 2017). Heritability of common disorders is caused by the polygenic effects of thousands of DNA variants, each accounting for infinitesimally small amounts of variance (Visscher et al., 2021). Similarly, nonshared environment might be due to many events with very small effect sizes. Detecting these ‘poly‐environmental’ effects will be much more difficult than identifying genetic effects for two reasons. First, heritability is limited to the effects of several million inherited differences in the three billion base pairs of DNA in the genome, but there is nothing comparable to the DNA code that can frame the search for environmental transmission. Nonetheless, it has been suggested that technological advances that can capture and analyse thousands of micro‐environmental events in real time—such as wearable devices and online footprints analysed by machine learning—offer a direction for research on the ‘environome’ (von Stumm & d’Apice, 2022). It is an empirical issue whether these new technologies can capture systematic environmental factors that predict nonshared environmental effects after controlling for genetics.

The second reason is that genetic effects are mostly additive, which is the genetic reason why children resemble their parents. Additive effects, even though extremely small, are possible to detect in genome‐wide association studies, whereas the power to detect nonadditive, interactive effects, especially higher‐order interactions among many genes (epistasis), is daunting. In contrast, there is no comparable reason to expect that environmental effects are constrained to be additive; that is, environmental effects could be entangled in high‐order interactions.

Note that all the categories of potential nonshared environmental influence in Table 1 are exogenous events, which reflects the traditional view of the environment as events ‘out there’ that are imposed passively on us. Instead of assuming that nonshared environmental effects are caused by random environmental events external to the individual, a more radical possibility is that these nongenetic effects emerge from random endogenous noise in biological systems independent of exogenous events.

## CLONES

Support for this possibility comes from decades of research on clones reared in laboratory conditions that control the environment. Dozens of inbred strains of mice, for example, have each been created by inbreeding mice for many generations so that individuals within a strain are essentially identical genetically like clones but different from other strains. Average differences between inbred strains from multiple litters are ascribed to their genetic differences on the assumption that their environments are uniform. These studies indicated widespread genetic influence on behavioural variability (Sprott & Staats, 1975). However, even more striking than these average differences between strains is the wide range of individual differences within inbred strains despite being reared in highly standardised laboratory conditions.

Because pups from inbred mouse strains are raised in the same litter with up to a dozen genetically identical siblings, environmental variance can be separated into variance between litters and variance within litters, which directly tests for shared and nonshared environment, respectively (Neiderhiser, 1989). As in human studies, this research suggests that environmental variance is almost entirely nonshared.

Differences within inbred strains have been observed for more than a century (Wright, 1920), but a 2013 article entitled ‘Emergence of individuality in genetically identical mice’ resurrected interest in this phenomenon (Freund et al., 2013). The authors reported individual differences in exploratory activity of genetically identical mice that increased with age. This article motivated more than a dozen studies along these lines, culminating in a series of studies on clonal fish, the Amazon molly, which reproduces asexually and requires no maternal care so that each genetically identical fish can be reared in its own individual tank with identical conditions (Laskowski et al., 2022). Despite these uniform environments and genes, reliable individual differences in swimming speed were present on the first day and predicted swimming speed 10 weeks later. Although these differences could be due to systematic prenatal effects, embryo splitting and transfer experiments have found no support for this hypothesis. For example, in mice, identical twins can be created by splitting embryos and these identical twin embryos can be implanted together in the uterus of one foster mother or implanted separately in uteri of different foster mothers (Gärtner, 1990). Developing in the same womb or different wombs had no effect on the similarity of these identical mouse twins, indicating that prenatal environments have negligible systematic effects.

## ENDOGENOUS NOISE

The most interesting implication of this clonal research is its suggestion that random factors responsible for nonshared environment might be endogenous, independent of exogenous events. Increasingly complex processes have been proposed: ‘random somatic effects’ (Fisher, 1918); ‘developmental irregularities’ (Wright, 1920), ‘intangible developmental variation’ (Falconer, 1960); ‘nonlinear epigenetic processes’ (Molenaar et al., 1993), ‘intrinsic chance variations’ (Finch & Kirkwood, 2000), ‘molecular noise of biological systems’ (Oates, 2011), ‘biological random variability’ (Gärtner, 1990), ‘nonlinear mechanisms during patterning and morphogenesis’ (Vogt, 2015), and ‘nonlinear inter‐molecular interactions involved in cellular function, coupled with thermodynamic instability’ (Honegger & de Bivort, 2018). These nonshared environmental candidates have been summarised as ‘the intrinsic stochasticity of molecular processes’ (Tikhodeyev & Shcherbakova, 2019), or even more succinctly, biological noise.

The apparent semantic contradiction of an endogenous process being a source of nonshared *environment* dissolves when it is understood that the term *nonshared environment* refers to nonshared environmental *effects*, not to environments external to the individual. These nonshared effects are only called environmental in the tradition of genetic research which puts genetics in the foreground and the environment refers to the residual variance in the background not explained by genetics; this residual variance is more accurately labelled *nongenetic*. Nonshared environment is the residual nongenetic variance not explained by shared environmental influence. The touchstone for nonshared environmental effects is whatever makes identical twins different, which includes processes that are nonsystematic as well as systematic, and endogenous as well as exogenous, in addition to error of measurement.

Any biological process can be considered as a source of differences within pairs of identical twins. For example, in his 1987 book *Neural Darwinism*, Gerald Edelman wrote about developmental noise at a neuronal level that ‘insures individuality—while identical twins may have closer neuroanatomic structures than outbred individuals, it is predicted that they will nonetheless be found to have functionally significant variant wiring’ (Edelman, 1987, p. 323). The probabilistic nature of neural development has often been discussed subsequently (e.g., Mitchell, 2018).

Although biological noise is unpredictable, we can assess its nonshared environmental associations with psychopathology after controlling for genetics. An efficient design to screen for stable nonshared environmental effects free of genetic influence is to assess them in identical twins who are discordant for psychopathology because reliable differences within pairs of identical twins can only be due to nonshared environment. However, just as with exogenous events, determining causal directions for the association between endogenous experiences and psychopathology is difficult. It is also challenging to demonstrate that the endogenous experiences are caused randomly.

Two examples of biological noise that could have nonshared environmental effects are somatic mutations and epigenetics.

### Somatic mutations

Conceptually, the best example of biological noise is somatic mutations because mutations in DNA sequence are random and causal in the directional sense that there is no backward causation. Somatic mutations are noninherited mutations that accumulate with each cell division, which leads to genomic mosaicisms within and between tissues. Although somatic mutations are relatively rare, they increase linearly throughout the life course, and there is a burst of somatic mutations prenatally that may be especially important because these are shared by many descendent cells (Blokzijl et al., 2016). Identical twin pairs differ on average by five early mutations (Jonsson et al., 2021).

Studies of postmortem brains show promising results linking somatic mutations with behavioural disorders. For example, brains of individuals with autism show an excess of somatic mutations in neural enhancer sequences (Rodin et al., 2021); increased mutations in single human neurons have also been associated with ageing and neurodegeneration (Rodin et al., 2021). A new $140‐million project, called Somatic Mosaicism Across Human Tissues, will create a body‐wide reference of somatic mutations using whole‐genome sequencing of 15 postmorte tissues from 150 healthy people (Leslie, 2023).

In vivo studies do not have access to brain tissue. Studies of discordant identical twins primarily using blood have had limited success in finding excess somatic mutations in the affected twin (Tazelaar et al., 2023). As the cost of whole‐genome sequencing continues to decline, it should be possible to relate somatic mutations to behaviour in large samples of unrelated individuals using multiple accessible tissues. If mutations differ across tissues, they can be assumed to be noninherited, which obviates the need for identical twins or other family members to exclude inherited mutations.

### Epigenetics

Any ‘‐omic’ processes along the pathways from genes to brain to behaviour can be investigated as a source of behavioural differences within pairs of identical twins. The methylome has received the most attention. Epigenetics is the study of mechanisms that change gene expression without altering inherited DNA sequence. It is generally assessed in blood as the quantity of RNA transcripts from DNA sites that are subject to a regulatory mechanism called methylation. Like somatic mutations, epigenetic changes in DNA methylation are tissue‐ and cell‐specific and accumulate during the life course. Unlike somatic mutations, epigenetic changes can be reversed. A study of identical twins reporting that the accumulation of differences in methylation across the genome (the methylome) correlates with phenotypic discordance (Fraga et al., 2005) sparked a dozen epigenetic studies on identical twins discordant for psychopathology (Castillo‐Fernandez et al., 2014).

Although the search for epigenetic signatures of these disorders has reported significant differences between discordant identical twins, effect sizes are small for individual RNA transcripts. Until recently (Odintsova et al., 2021), there has been nothing in epigenetics equivalent to the polygenic scores used in genomic research that aggregate effects across thousands of genetic associations. Nonetheless, the low average identical twin correlation (0.14) for individual differences in hundreds of thousands of methylation sites in twin studies of DNA methylation (Li et al., 2022) suggests that further research is warranted on epigenetics as a source of nonshared environmental influence.

#### Idiographic approach

Research on identical twins discordant for psychopathology has mostly been nomothetic, looking for average effects in the population. Although multiple random processes can have stable effects on an individual, these multiple random processes are unlikely to have the same pattern of effects across individuals. For this reason, an idiographic approach may be needed to investigate idiosyncratic patterns of differences within each pair of identical twins. The studies of identical twins discordant for psychopathology is an example of the richness of an idiographic approach, for example, for major depressive disorder (Kendler & Halberstadt, 2013) and schizophrenia (Gottesman & Shields, 1972).

However, at the level of biological noise, if unique patterns of differences were found within each pair of discordant identical twins, it will be difficult to go beyond acknowledging the causal complexity of nonshared environmental effects. Nonetheless, it is possible that the power of machine learning, and artificial intelligence more generally, can be harnessed to extract nomothetic patterns from idiographic data from samples of identical twins discordant for psychopathology.

#### Change as well as continuity

Although research on inbred strains and clones highlights processes that begin prenatally and contribute to stability, random processes, whether endogenous or exogenous, can be investigated as sources of change, not just continuity. For example, dynamic endogenous processes such as gene expression and epigenetics can be investigated longitudinally to assess their contribution to age‐to‐age changes in psychopathology within individuals. The discordant identical twin design is not suitable for idiographic analyses of change because the design begins with the assumption of stable differences within cotwins, such as identical twin pairs selected for discordance for psychopathology. Idiographic patterns of change across time as they affect age‐to‐age change in symptoms of psychopathology can be investigated within individuals as a first step towards identifying dynamic sources of nonshared environmental effects. Although genetic effects on such change seem unlikely, they cannot be ruled out until they are tested, for example, within identical twin pairs. If change were studied across brief time intervals, these random processes might account for some of what we call error of measurement.

The contribution of biological noise to change as well as continuity is important because longitudinal research on psychopathology and personality in human development indicates that correlations between nonshared environmental effects across 5‐year windows in adulthood are about 0.50, indicating that they contribute equally to change and continuity (Briley & Tucker‐Drob, 2014). Although there are no comparable meta‐analyses for psychopathology, similar results were found in a longitudinal twin study of attention problems (Kan et al., 2013) and in longitudinal studies of identical twins for externalising problem symptoms (Burt et al., 2009) and anxiety and depression symptoms (Kendler et al., 2011). A twin study of diagnosed depression from age 28 to 38 found that nonshared environment was responsible for change (59%) more than continuity (17%) (Bjørndal et al., 2023). The point is that nonshared environmental factors are involved in change as well as continuity.

#### Psychological noise

Similar to biological noise, the complexity of psychological processes can be viewed as an endogenous source of noise that contributes to nonshared environmental effects independent of exogenous events. For example, the current neuroscience model of consciousness suggests that we live in unique perceptual worlds (Seth, 2021). We do not see the world as it is—the brain is in a box and does not see or hear anything directly. In this view, the brain is a Bayesian prediction machine that minimises prediction error, creating our conscious reality from neural information from our eyes and ears. In this sense, perceptions as well as thoughts and feelings are controlled hallucinations. This complexity leads to the proposition that our perceptual worlds are idiosyncratic. In other words, nonshared environmental effects could be caused by individual differences in such random (unpredictable) endogenous psychological experiences rather than exogenous events. To highlight the distinction between endogenous experience and exogenous events, this source of nonshared environmental influence could be called *nonshared experience.*

Nonshared experience might be especially tractable for understanding nonshared environmental effects using an idiographic approach. For example, the results of research on discordant identical twins can be interpreted as showing the importance of subjective nonshared experiences such as romantic relationships (Kendler & Halberstadt, 2013), social support (Crush et al., 2020), and perceived vulnerability (Asbury et al., 2017) that could originate in random endogenous psychological experiences.

Kendler and Halberstadt's concluding remark about their intensive interviews with identical twins discordant for depression is especially insightful: ‘This was a humbling experience. An individual's personal experiences are an entwined mix of how they construct their world and what that world does to them outside of their control’ (Kendler & Halberstadt, 2013, p. 980).

## IMPLICATIONS

Genetic research provides the best available evidence for the importance of environment—it accounts for the other half of the variance not explained by genetics—but it is not the environment shared by children growing up in the same family, living in the same neighbourhood, and attending the same school. The reality of nonshared environment is not just relevant to understanding why siblings in the same family differ; it is how the environment affects the development of individual differences for all children. Recognition that nonshared environment is real has far‐reaching implications for scientists, clinicians, parents, and for understanding ourselves, as discussed elsewhere (Plomin, 2019).

Just as far‐reaching are the implications of recognising that nonshared environmental effects are random, that is, unpredictable. Three decades of research have not revealed systematic sources of nonshared environment for psychopathology. Although the absence of evidence is not evidence of absence, it is time to take seriously the possibility that random, unpredictable factors are responsible for nonshared environmental effects. These random factors are not limited to exogenous events such as accidents and illnesses. Research on clones raised in identical environments and the pervasiveness of randomness in biological systems indicates that endogenous processes can generate developmental differences within pairs of identical twins. Biological noise has been considered in relation to nonshared environment, but noise emanating from the complexity of psychological processes could also create nonshared environmental experiences. Endogenous processes such as somatic mutations and epigenetics can be investigated as sources of nonshared environmental effects, not just as mediators of exogenous processes.

Randomness is fundamental to physics and all biological systems, as argued cogently by Jacques Monod in his 1979 book, *Chance and Necessity* (Monod, 1979). In physics in the 1920s, particle‐wave duality and the uncertainty principle led Neils Bohr to conclude that randomness and probability must replace determinism at the core of quantum mechanics, although Albert Einstein persisted to the end in believing that randomness is merely a sign of our ignorance of a deeper level of deterministic causation (‘God does not play dice with the universe’). A recent book called *The End of Everything (Astrophysically Speaking)* describes randomness from quantum mechanics to cosmology, from the unimaginably small of quantum mechanics to the unimaginably large of cosmology (Mack, 2020).

Scores of book‐length treatments of randomness have been written for diverse fields beyond physics and biology, such as philosophy (Rescher, 1995), mathematics (Beltrami, 2020), economics (Frank, 2016; Kay & King, 2020), gerontology (Finch & Kirkwood, 2000), and sports (Smith, 2012). A lively popular book called *A Series of Fortunate Events: Chance and the Making of the Planet, Life, and You* (Carroll, 2020) provides an overview of randomness of life at the level of cosmology (asteroids hitting earth), geology (tectonic plate shifts), and biology (DNA mutations), concluding that:

‘We are all here, both collectively and individually, through a series of accidents—cosmological, geological and biological accidents. Our chance‐driven world is a profound revelation. It is astonishing that blind chance is the source of all novelty, diversity, and beauty in the biosphere’ (p. 164).

The most general scientific implication is the need to transition from determinism to a probabilistic view that gives randomness its due, as discussed in a book called G*enetics and Randomness* (Ruvinsky, 2017), which concludes:

‘The major aim of science is finding general principles, laws, and mechanisms, as well as regular and predictable events. Randomness, at first glance, is the alternative to all of these and may appear to be an annoying nuisance that limits science and prevents generalizations. Such a deterministic and outdated understanding of science is steadily being replaced by the probabilistic view which became so typical for genetics’ (p. xiii)…. ‘such epistemology does not indicate a potential crisis in science and does not look gloomy’ (p. 14).

This view has been applied specifically to nonshared environment in an article, *The ‘gloomy prospect’: embracing randomness*, which ends with a section called *Learning to live with randomness* (Davey Smith, 2011). Although this article was aimed at epidemiology with its emphasis on group‐level rather than individual‐level analysis, most of its points are also relevant to psychology and psychopathology. The issue is this: if you could rewind the clock and replay the events, would things play out the same? Although that experiment cannot be run, identical twins growing up in the same family approach this counterfactual, and their data indicate that the answer is no.

Nonetheless, randomness limits our ability to predict. Finding that nonshared environment is random implies that psychopathology is unpredictable environmentally. In other words, attempts to unravel the environmental origins of psychopathology are not likely to be productive despite the centrality of the psychoanalytic assumption that understanding the historical (environmental) causes is prerequisite to curing current problems. However, causes and cures are not necessarily related. Recognising random endogenous or exogenous causes of psychopathology does not restrict attempts to ameliorate these behavioural problems. Similarly, prevention is not necessarily related to understanding causes. For example, mindfulness training can be employed to buffer all children against sliding into cognitive perceptions and ruminations that lead to depression even if the actual environmental tipping points for depression are random endogenous nonshared processes. Moreover, understanding the biological and psychological processes by which these random causes have their effect could lead to more personalised treatments.

Randomness also limits genetic influence, resulting in heritabilities of 50% rather than 100%. Viewed from the perspective of biological and psychological noise, it is amazing that inherited differences in DNA sequence in the single cell with which life begins can have such systematic effects across the trillions of cells in the human body throughout the life course. As polygenic scores get closer to their predictive ceiling of heritability, they will be used to predict psychopathology from birth and thus facilitate prevention (Plomin, 2022). However, these predictions will always be probabilistic, limited by heritabilities of about 50%.

Even in the case of genetics, knowing that a child diagnosed with Attention Deficit Hyperactivity Disorder (ADHD) has a high polygenic score for ADHD does not as yet alter planning for cognitive, behavioural or pharmacological treatments to control the child's attentional problems and hyperactivity. However, personalised treatment and especially prevention are likely to come from genetics, which can already predict psychopathology better than measured environments. Even though polygenic score analyses of psychopathology were first constructed only a decade ago, they can predict on average 4% of the liability for psychopathology, ranging from 1% for anxiety and anorexia to 8% for bipolar depression (Plomin, 2022). Moreover, a polygenic score analysis of behaviour problems comparable to the Gidziela et al. (Gidziela, Malanchini, et al., 2022) analysis of nonshared environment concludes that ‘multi‐GPS prediction of behaviour problems increased from <2% of the variance for observed traits to up to 6% for cross‐age and cross‐rater composites’ (Gidziela, Rimfeld, et al., 2022).

Because nonshared environment is the way the environment influences psychopathology, it is important to understand how it works even if we cannot predict its effects. Regardless of whether this article has been persuasive in arguing that nonshared environment is real but random, I hope it will help resurrect interest in trying to understand the ‘other’ 50% of the variance in psychology and psychiatry.

## AUTHOR CONTRIBUTIONS

**Robert Plomin**: Conceptualization; Funding acquisition; Investigation; Methodology; Writing – original draft; Writing – review & editing.

## CONFLICT OF INTEREST STATEMENT

The author has declared that they have no competing or potential conflicts of interest.

## Ethical considerations

Ethical approval was not required for this review.

## Data Availability

Data sharing is not applicable to this article as no datasets were generated or analysed during the current study.
